# Mapping Methods: Charting Future Priorities for Research Methods in Dissemination and Implementation Science

**DOI:** 10.21203/rs.3.rs-8874640/v1

**Published:** 2026-04-29

**Authors:** Stephanie Mazzucca-Ragan, Ross C Brownson, Sara Malone, Douglas A Luke

**Affiliations:** Washington University in St. Louis; Washington University in St. Louis; Washington University in St. Louis; Washington University in St. Louis

**Keywords:** dissemination and implementation (D&I) research, concept mapping, research methods, evidence-based programs

## Abstract

**Background:**

Closing the knowledge-practice gap requires applying research methods to advance the impact of dissemination and implementation (D&I) science. The purpose of this study was to identify research methods that are key to closing the research-practice gap, as determined by D&I researchers.

**Methods:**

The study used Concept Mapping—a mixed, participatory method that uses a structured approach to solicit and organize group opinions about a specific conceptual area. Ninety-eight researchers with expertise in D&I science (e.g., those associated with D&I research centers, training programs) were invited to participate in the study via the GroupWisdom online platform. Participants first completed the brainstorming prompt: “To accelerate the dissemination and implementation of evidence-based chronic disease prevention and control programs and policies, the most important research methods are…” Statements were reviewed for clarity and de-duplicated. Participants then sorted statements into similar groupings and rated statements according to 1) how important they are to D&I research and 2) how frequently they are currently used. The research team identified a cluster map depicting groupings of research methods derived from multidimensional scaling and cluster analysis of the sorted data.

**Results:**

Twenty-six participants generated 117 statements that were reduced to 89 after duplicates and extraneous statements were removed. Twenty-eight respondents sorted and rated those 89 statements. Twelve clusters were identified: theoretical and conceptual approaches; designing for dissemination; documenting and adapting implementation; measurement and evaluation; mechanism and cost analyses; user-centered design; partnerships and co-creation; analytic methods; systems and computational approaches; study designs; applied and adaptable methods, and rapid cycle methods. Participants ranked statements in the *user-centered design* cluster highest in importance, whereas *designing for dissemination* and *analytic methods*were ranked lowest in importance. *Study designs* was ranked the highest in frequency of use, while *designing for dissemination, mechanism and cost analyses*, and *systems and computational approaches* were ranked the least frequently used.

**Conclusions:**

These findings highlight clear priorities for methods development in D&I research, particularly in areas that are viewed as essential but remain underused. Aligning training, funding, and methodological innovation with these priorities can accelerate the rigor, relevance, and impact of D&I science.

## INTRODUCTION

Dissemination and implementation (D&I) science seeks to facilitate the adoption, adaptation, and sustainment of evidence-based programs and policies in routine clinical and public health settings (1). As a result, D&I science is inherently impact-oriented, emphasizing relevance to real-world contexts, engagement with end users, and outcomes that reflect meaningful change in practice and population health (2). Achieving these goals places distinct demands on the selection and use of research methods. D&I studies are often conducted in complex, dynamic systems and require approaches that can account for multilevel influences, contextual variation, and equity considerations, all while balancing rigor with feasibility. Becker and colleagues articulated several gaps that D&I research seeks to address, including gaps in public health supply and demand, expertise and capacity, and the availability of research methods capable of addressing these challenges gaps (3). Among these, methodological gaps are particularly consequential, as the ability of D&I research to generate actionable evidence depends on the availability and appropriate use of methods suited to pressing implementation problems.

Many research methods have been developed and applied to support D&I research, including experimental and quasi-experimental designs, mixed-methods approaches, multilevel measurement and analysis, and stakeholder-engaged research practices (4). These foundational methods have enabled important advances in understanding determinants of implementation and evaluating the effects of implementation strategies on relevant outcomes (5–8). However, as the field evolves, D&I research questions increasingly extend beyond whether implementation works to how, why, for whom, and under what conditions it works, as well as how interventions can be adapted, scaled, and sustained equitably and efficiently (6, 9). Addressing these questions requires continued refinement of existing methods and the development and uptake of additional methodological approaches. At the same time, D&I science draws on multiple disciplinary traditions, creating opportunities to leverage advances from fields such as epidemiology, systems science, economics, and improvement science, while also increasing the complexity of the methodological landscape.

Simultaneously, multiple efforts have highlighted the need for continued methods development to support the evolving goals of D&I science. Prior work has identified gaps in methodological training, competencies, measurement, and analytic approaches, as well as the need for methods that better support relevance to practice and policy contexts (10–13). Despite this growing recognition, existing efforts have largely identified broad areas of need rather than providing an empirical assessment of how D&I researchers prioritize specific methodological approaches relative to their current use. Together, these trends underscore the need for a clearer understanding of which methods are viewed as most important for advancing D&I research and where gaps remain between methodological priorities and current practice. It remains unclear which methods are perceived as most critical for advancing the field, which are already sufficiently developed and integrated into routine practice, and which represent underutilized opportunities for methodological innovation or dissemination. Without this information, it is difficult for researchers, funders, and training programs to strategically target investments in further research methods development and capacity building.

Concept mapping is a structured, participatory mixed-methods approach that integrates qualitative idea generation with quantitative organization and prioritization (14, 15). Through a multistep process, participants generate statements in response to a focused prompt, sort those statements into conceptually meaningful groups, and rate them on predefined dimensions such as importance or feasibility. The resulting concept maps visually and statistically represent shared understanding within a group, while preserving diversity of perspectives. Concept maps have been used to great effect in public health and D&I research (11, 16–21). Its ability to capture both consensus and variation makes it particularly well suited for examining methodological priorities in D&I research, where expertise spans multiple disciplines and methods are applied across diverse settings.

The purpose of this study was to use concept mapping to identify, organize, and prioritize research methods needed to advance D&I science. This approach enables the identification of areas where methods are widely used and well established, as well as areas where methods are viewed as highly important but used less frequently, suggesting opportunities for targeted development, dissemination, and training. Methods that will support rigorous, pragmatic, and equitable research. By providing an empirically grounded framework of D&I research methods and highlighting gaps between methodological priorities and current practice, this study offers a roadmap to ensure researchers can conduct rigorous, pragmatic, and equitable D&I research with meaningful real-world impact.

## METHODS

This study identified D&I research methods needs through concept mapping, which is a mixed-methods, participatory approach to gathering and organizing ideas through several steps: develop the focus prompt; identify participants; generate ideas; structure ideas; analyze data and create visual representations; and interpret and use results (14). Groupwisdom (formerly, Concept Systems Global MAX), a web-based software, was used through all steps of the process. This study was approved by the Washington University in St. Louis Institutional Review Board (IRB# 202004114).

### Develop a focus prompt.

The study team developed the focus prompt based on the goals of the study and input from colleagues who have conducted or participated in concept mapping studies, including members of the WashU D&I collaborative (22). The final focus prompt was *“to accelerate the dissemination and implementation of evidence-based chronic disease prevention and control programs and policies, the most important research methods are…”*

### Identify the participants.

A multi-step process was used to identify participants. Through publicly available listings, we identified leaders in D&I research from their participation as a(n)

- Principal Investigator of the National Cancer Institute-funded Implementation Science Centers for Cancer Control
- Director of a Centers for Disease Control and Prevention-funded Prevention Research Center
- Editor of *Implementation Science* or *Implementation Science Communications* journals
- Plenary or keynote presenter at the Annual Conference on the Science of Dissemination and Implementation in Health
- Author of a book chapter for 2^nd^ edition of *Dissemination and Implementation Research in Health: Translating Science to Practice*
- First or last author of a methodology article in *Implementation Science*
- Faculty or mentee in national training programs (i.e., Mentored Training for Dissemination and Implementation Research in Cancer, Implementation Research Institute, Mentored Training in Implementation Science)
- Member of the National Institutes of Health Science of Implementation in Health and Healthcare study section
- Member of the National Cancer Institute Implementation Science group
- Leader in the Society for Implementation Research Collaboration

From these groups, 659 researchers were identified. To reduce the number of potential participants to a reasonable number, we identified those who fell in two or more of these categories (n=144). Limiting the sample in this manner also increased the proportion of international and early- and mid-career researchers relative to the initial list.

### Generate ideas through brainstorming.

Individuals were invited to the study via email, which directed them to the Groupwisdom project site. A random sample of 100 participants were invited from the 144 individuals identified. Participants accepted a written consent statement in the software and then were asked to complete the focus prompt statement with as many statements as they desired. All previously generated statements were visible to participants, and statements were anonymous.

Statements were edited for clarity and grammatical correctness by two members of the research team (SMR, DAL). Statements containing multiple ideas were split into two items so that only one idea was present in each statement. Duplicative statements were removed. The statement list was finalized in alignment with concept mapping best practices, which recommend limiting the sorting and rating phases to fewer than 100 unique statements (15).

### Structure ideas through sorting and rating.

After the brainstormed statements were compiled into a list of unique statements, participants who completed the brainstorming step were invited to sort the statements based on how they perceived the statements to be related. They could create as many or as few groupings (piles) as they saw to be a good fit for the statements, similar to a card sorting activity. Each statement could appear in only one pile, and no statement could be sorted into its own separate pile. Participants were asked to name each pile to represent all statements in that pile.

Then, participants were asked to rate the statements based on two criteria, 1) importance of the method (*How important is each method to the field of dissemination and implementation science?)* from 1 “not at all important” to 7 “very important” and 2) how common the method is used (*How often has or is each method been used in dissemination and implementation research in the past or present?)* from 1 “not at all common” to 7 “very common”.

### Gather information about participants.

At the sorting and rating phase, participants were asked demographic questions, including 1) *Which do you consider your level of dissemination and implementation research expertise to be?* (Beginner, Intermediate, Advanced); 2) *How many years of experience do you have in your field?* (<5 years, 5–9 years, 10+ years); and 3) *What do you consider your level of research methodology expertise to be?* (Beginner, Intermediate, Advanced).

### Analyze and visualize the data.

A cluster map was created to summarize the sorting activity among all participants. The Groupwisdom platform creates this map using several quantitative methods. First, a similarity matrix is created to consolidate the sorting data from all participants. Then, multidimensional scaling is used to create a point map in which each statement is represented as a point on a two-dimensional plane (23, 24). Distances between points reflect how often statements were sorted together; points that are closer together are interpreted as more conceptually similar. Hierarchical cluster analysis is used to group points together that represent similar concepts (25, 26). The research team examined cluster maps of four to 15 cluster solutions to determine which best reflected the point map and were most interpretable.

Each cluster map was evaluated for the consistency of ideas within a cluster and for the bridging values of statements and clusters, which indicate how often statements were sorted together. Lower bridging values (ranging from 0 to 1) indicate that statements within a cluster were commonly sorted together. Also, the cluster solution’s stress index was considered, which indicates the goodness of fit of the cluster solution (i.e., a lower stress index indicates a better overall fit).

Ratings of each statement were averaged, and an overall cluster average was calculated. A ladder graph was produced in Groupwisdom to visualize and compare the cluster-level averages for the importance and use ratings. Additionally, a “Go-Zone” map was created, which maps each statement to one of the two ratings to create a priority-quadrant plot to guide which statements should be the focus of future methods work.

## RESULTS

The research team invited 100 participants to the brainstorming portion of the study, during which 26 participants generated 117 statements. After reviewing the initial list of brainstormed statements, we reduced the statements to 89 unique statements. Some statements were broken into separate ideas, for example “Longitudinal study designs and analytic methods” was broken into “Longitudinal study designs” and “Longitudinal analytic methods.” Other ideas consolidated from multiple reports to a single instance; for example, “mixed methods” appeared in seven of the initial statements.

Twenty-eight participants completed the sorting and two rating activities. Characteristics of these participants are in Table 1. Approximately 36% were affiliated with Washington University, about half were based elsewhere in the United States, and 18% were based internationally. Participants ranged in their years of experience and in the rating of their D&I research expertise, with most rating themselves as advanced in D&I research (43%). More than 80% of participants rated themselves as intermediate or advanced experts in research methods.

### Clusters of D&I research methods

**Figure 1:**
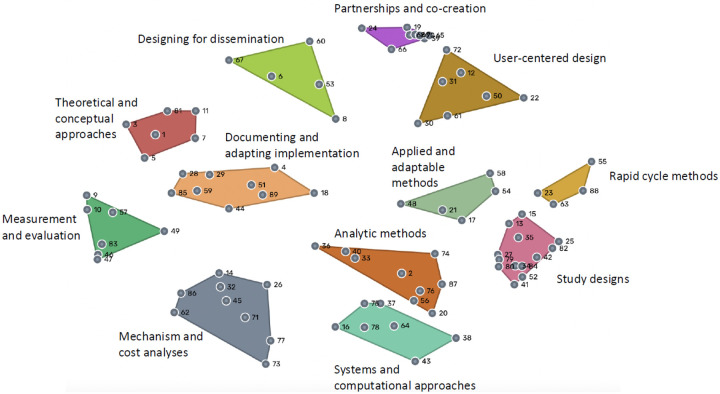
12-cluster concept map of prioritized research methods for dissemination and implementation science

A final, 12-cluster map was identified, with clusters containing between four and 13 statements: 1) *theoretical and conceptual approaches*, 2) *designing for dissemination*, 3) *documenting and adapting implementation*, 4) *measurement and evaluation*, 5) *mechanism and cost analyses*, 6) *user-centered design*, 7) *partnerships and co-creation,* 8) *analytic methods,* 9) *systems and computational approaches*, 10) *study designs*, 11) *applied and adaptable methods*, and 12) *rapid cycle methods* (Figure 1). The stress value of the final map was 0.2813, which falls between typical values for concept mapping studies (0.205 – 0.365) (14). Stress values indicate how well the map represents the sorting data, with lower values indicating better fit. which falls within typical ranges for concept mapping studies (14). Table 2 lists each sorted item by cluster, along with their rating scores.

Similar clusters are shown near each other, whereas clusters farther from each other are less similar. We organized the clusters into four higher-order groups based on their physical proximity on the cluster map, reflecting conceptual similarity. Three clusters described the conceptualization and development of implementation strategies. The *theoretical and conceptual approaches* cluster included methods using theories, frameworks, and logic models to conceptualize D&I problems and guide implementation strategies. The *designing for dissemination* cluster highlighted methods that intentionally design evidence and strategies to enhance dissemination and uptake across settings. The *documenting and adapting implementation* cluster focused on methods to document, examine, and adapt implementation strategies across contexts and over time.

Four clusters focused on measurement and analytic methods. The *analytic methods* cluster comprised methods for analyzing complex, multilevel, and longitudinal data to assess implementation processes, outcomes, and disparities. The *measurement and evaluation* cluster captured methods for developing and applying measures of implementation outcomes and organizational context using diverse data sources. The *mechanism and cost analyses* cluster included methods to test implementation mechanisms and evaluate mediators, moderators, costs, and cost-effectiveness. The *systems and computational approaches* cluster captured methods to model complex systems and understand implementation across interconnected structures and contexts.

Three clusters emphasized real-world relevance, external validity, rapid learning, and application of D&I research in practice settings. The *rapid cycle methods* cluster included methods to support rapid, iterative learning and continuous improvement during implementation efforts. The *applied and adaptable methods* cluster comprised methods emphasizing external validity, scaling, and long-term implementation in organizational settings. The *study designs* cluster represented qualitative, quantitative, and mixed-methods designs to examine D&I processes, outcomes, and context.

Last, two clusters highlighted stakeholder-centered and participatory approaches. The *partnerships and co-creation* cluster included methods to work collaboratively with those outside of academia methods engaging patients, providers, policymakers, and communities through participatory and co-creation approaches. The *user-centered design* cluster comprised methods to develop pragmatic, culturally appropriate implementation strategies informed by the needs and perspectives of intended users.

### Importance and Frequency Ratings of Clusters and Statements

**Figure 2:**
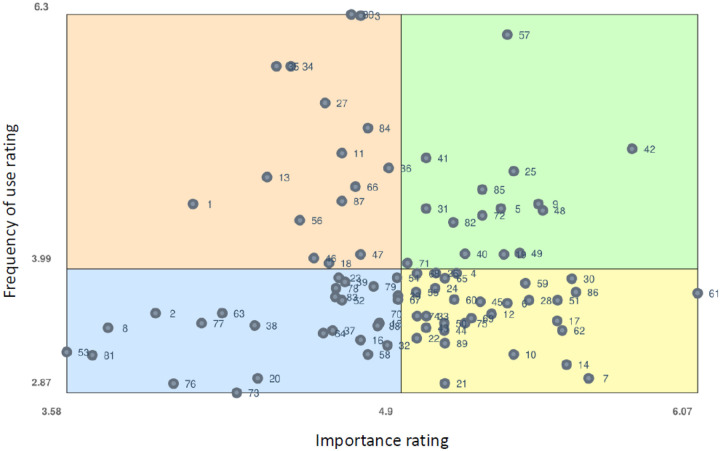
Ladder graph illustrating alignment between perceived importance and frequency ratings of dissemination and implementation research methods.

Figure 2 shows the average importance and frequency ratings across the 12 clusters. The variability of average cluster-level ratings for importance of the methods (4.53 to 5.31) and frequency of use (3.49 to 4.92) across the 12 clusters was modest, and importance was rated higher than frequency of use for all clusters except *study designs*. *User-centered design* was ranked the highest for importance, and *designing for dissemination* and *analytic methods* were ranked the lowest for importance. *Study designs* was ranked the highest for frequency of use, while *designing for dissemination*, *mechanism and cost analyses*, and *systems and computational approaches* were ranked the least frequently used. The three clusters with the largest difference between the importance and frequency of use ratings were *applied and adaptable methods*, *mechanism and cost analyses*, and *user-centered design*.

**Figure 3:**
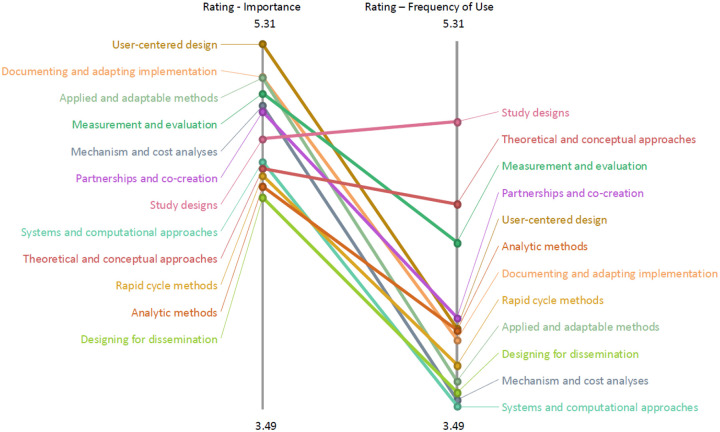
Quadrant map of implementation research methods based on perceived importance and frequency ratings

A priority-quadrant graph was created by plotting statements by their average importance on the x axis and frequency of use on the y axis and grouping statements above and below the mean ratings on each axis, creating four quadrants (Figure 3). The high importance/low frequency (yellow) quadrant highlighted statements that researchers view as priorities for methods development. Statements in this quadrant focused on designing for dissemination, documenting and adapting implementation strategies, measuring and testing mechanisms, economic evaluation, equity-focused approaches (noted as disparities), rapid-cycle learning embedded in practice, scaling and sustainment, and co-creation with stakeholders. In contrast, the high importance/high frequency (green) quadrant included foundational D&I methods, such as multilevel and mixed-methods designs, measurement and evaluation of implementation outcomes, assessment of organizational context, and stakeholder-engaged and practice-based research. The low importance/high frequency (orange) quadrant largely reflected traditional designs, frameworks, and analytic approaches. The low importance/low frequency (blue) quadrant reflected more specialized or resource-intensive, such as systems science, advanced causal inference, and computational modeling.

Clusters with the most statements in the priority quadrant (yellow, high importance/low use) were *documenting and adapting implementation* (n=6), *mechanism and cost analyses* (n=5), and *user-centered design* (n=5). Example statements from these clusters are “Measuring adaptations of implementation and intervention strategies,” “Methods to test mechanisms of implementation strategies,” and “Methods involving user or human-centered design approaches.” The *study designs* cluster had no statements in the priority quadrant.

The clusters with the most statements in the green quadrant (high importance/use) were *study designs* (n=4) and *measurement and evaluation* (n=3). These clusters included statements such as “Multilevel designs” and “Methods that capture organizational context.” Statements in the orange quadrant (low importance/high use) were mostly those in the *study designs* cluster (n=6), for example “Randomized controlled trials and variations thereof.” Statements in the *systems and computational approaches* cluster were the most common in the blue quadrant (low importance/use, n=5 of 7 statements), for example “Systems science methods that capture heterogeneity, social structure, and system dynamics.”

## DISCUSSION

This study offers a structured, expert-informed assessment of current and emerging D&I research methods, highlighting both methodological strengths and gaps between perceived importance and use. We identified 12 method clusters organized into four domains spanning implementation strategy development, measurement and analysis, rapid and practice-relevant approaches, and stakeholder-centered methods. Foundational methods, such as multilevel, mixed-methods, and stakeholder-engaged approaches, were rated as widely used and highly valued. Methods considered essential for advancing the impact of D&I research but used less frequently included adaptation, mechanism testing, economic evaluation, designing for dissemination, and sustainment and scale-up. To our knowledge, this is the first empirical study of methodological gaps and priorities for D&I research. These findings provide clear direction for prioritizing methods development and capacity building to support more rigorous, pragmatic, and equitable D&I research.

Our mapping of methods priorities can be compared with several other efforts—in combination, this provides a rich array of future research opportunities. For instance, Tabak and colleagues identified the need for new methods and measures as a main priority for D&I training programs (11). The top-rated cluster in Tabak et al was to make research more relevant, where several methodological issues are prominent, including designing for dissemination and documenting the impacts of de-implementation. In a recent survey of 97 D&I experts, Brownson and colleagues identified advanced skills that should be prioritized in training programs (10). Several findings align with the current study, including a focus on adaptation, sustainability, multilevel measurement, mechanisms, and designing for dissemination. Additionally, Emmons and colleagues described a modified version of the Translational Science Benefits Model that provides a set of metrics for mapping D&I methods in future work (27). Finally, many of the priorities emerging from this work align with current funding priorities for the US NIH (https://grants.nih.gov/grants/guide/pa-files/PAR-25-144.html).

Clusters ranked higher in importance but lower in frequency of use are groups of methods that need further development, better dissemination to D&I researchers, or both. The use of these methods may be limited by methodological complexity, limited training opportunities, lack of standardized measures or procedures, or misalignment with funding and academic incentive structures. Methods in three clusters – *documenting and adapting implementation*, *mechanism and cost analyses*, and *applied and adaptable methods* – were commonly found in the high-importance/low-frequency quadrant. Adaptation, grouped within the *documenting and adapting implementation* cluster, has long been a focus of D&I research (28–31). Conceptual work has focused on framework development (32, 33), and more recently, pragmatic measures and tracking methods have been developed (34–36). Adaptive study designs such as multiphase optimization strategy (MOST) and sequential, multiple assignment randomized trials (SMART) have been used in D&I (37), but they are complex and require advanced training to use. Additionally, the growing focus on mechanisms moves the field beyond describing whether implementation works to understanding how and why it works (38–40). Recent work has emphasized explicit specification of implementation strategies, their hypothesized mechanisms, and their link to theories, models, and frameworks (41, 42). Last, cost and cost-effectiveness are essential for long-term implementation success. Funders and decision-makers increasingly expect implementation studies to address affordability and return on investment (43, 44). Future work should focus on integrating economic evaluation into hybrid and pragmatic designs, standardizing cost measurement, and linking costs to mechanisms and outcomes. The next steps for each of these methodologic areas are distinct, but they should all build on developments in other fields, for example, mechanisms work has drawn on causal inference methods from epidemiology (45) and work in public health intervention science and clinical psychology to understand behavior change mechanisms (46).

Many of the clusters that had above average importance and frequency of use ratings are foundational to D&I science and represent the core values of the field. These clusters include methods that focus on community partners (*partnerships and co-creation* and *user-centered design*) (47, 48) and *measurement and evaluation* (5). The prominence of these clusters suggests consensus about their central role in conducting rigorous and relevant D&I research. Continued refinement is needed to ensure that engagement approaches meaningfully address issues of equity and power, and that measurement and evaluation methods are responsive to increasingly complex, adaptive, and time-sensitive implementation contexts. Strengthening these foundational methods will be critical to supporting the more advanced research questions and methodological approaches highlighted elsewhere in the concept map.

Methods that were rated as lower in importance but higher in frequency of use reflect approaches are sufficiently developed to meet the needs of most D&I research questions. These clusters, *theoretical and conceptual approaches, study designs,* and *analytic methods*, represent approaches that are well established, widely taught, and routinely applied across D&I studies. The lower importance ratings for these clusters should not be interpreted as a lack of value. Rather, they likely indicate that these methods are viewed as essential or expected components of rigorous D&I research, requiring less immediate attention for further development relative to other, cutting-edge methodological areas. These findings suggest that while continued use and appropriate application of established designs, frameworks, and analytic approaches remain critical, future methods development efforts in D&I science may be more productively focused on areas where methodological gaps persist rather than on approaches that are already well integrated into the field.

Clusters that were rated lower in importance and frequency of use included *designing for dissemination, rapid cycle methods*, and *systems and computational approaches*. Lower ratings may reflect limited familiarity with the benefits of these methods to D&I research, perceptions of narrower applicability within D&I research, or concerns about the intensity of resources required to use these methods. Next steps for these methodological areas should address the specific reasons researchers perceived them to be less important and use them less frequently. In the case of designing for dissemination, methods explicitly focused on dissemination planning and tool development remain underused relative to their perceived importance, though the principles of designing for dissemination have been present in D&I science since its inception (49). Resources to support researchers’ use of these methods have been developed, including the D4DIS webtool (50) and the D4D&I Learning Hub (51), which can promote broader uptake of designing for dissemination methods.

Although used less frequently, systems science methods are well suited to addressing several of the priorities identified in this study (52–56), in particular for identifying and testing mechanisms of change (57). Trainings, such as the Systems Science for Social Impact have been developed to help researchers understand how systems science methods fit within their research and how to get started using these methods (58). It will be important to address the unique challenges to using systems science methods in D&I research, for example how these methods align with common grant mechanisms and advanced training opportunities (59).

The heterogeneity of the concepts represented in the clusters underscores the interdisciplinary, team science nature of D&I research (60, 61). There are many types of expertise needed for the field to maximize the impacts of D&I efforts, and no single investigator can master all these methodological areas. These results reinforce the importance of team science for D&I research and the need for funding and institutional supports to facilitate team science. Also, the heterogeneity illustrates that D&I researchers conceptualize “methods” as a wide-ranging concept, including methods to design, test, and scale programs and policies. Some statements that were brainstormed do not fall neatly within standard definitions of research methods focused on methods used for data collection and analysis (62). Participants included ideas that represent a broad, system-level model of research. Tools to plan and design research (e.g., logic models and frameworks), processes for implementing research (e.g., co-design and advisory boards), and paradigms for conducting research (e.g., user-centered design) were represented in the statements brainstormed, in additional to traditional research methods. The broader conceptualization of research methods may reflect the somewhat blurry line between D&I research and D&I practice or the interdisciplinary nature of the field. Regardless, the ideas in the concept map represent what D&I researchers need to maximize the impact of their work.

The results of this study highlight priorities for the field moving forward. The concept map provides evidence that D&I science is maturing beyond a primary focus on identifying determinants of implementation or testing the effects of discrete implementation strategies on implementation outcomes. This next phase of D&I science builds on these foundational contributions to address the complexity, processes, and contexts of D&I, with increased attention to equity and the pace of implementation outside of research settings.

Advancing this phase of the field will require intentional investment in methods development. The methods prioritized by participants extend beyond traditional empirical techniques to include approaches that support complex, adaptive, and equity-oriented research questions. Development and dissemination of these methods may take multiple forms, including dedicated funding mechanisms, methodological papers, and methods studies embedded within substantive D&I studies. Training programs for doctoral students, postdoctoral trainees, faculty, and research staff should explicitly teach underutilized but high-priority methods, as well as the skills needed to apply them in partnership-based and practice-embedded research contexts.

The structure of the concept map also suggests a need for greater conceptual clarity within D&I science. The presence of both broad and narrowly defined clusters indicates areas where constructs, approaches, or tools may be inconsistently defined or overlapping, pointing to opportunities for refinement and shared terminology. Finally, these results reinforce the inherently interdisciplinary nature of D&I research. Methods development in D&I science should draw from advances in complementary fields rather than duplicating them.

The key strength of this study is the use of concept mapping, a participatory method that uses both quantitative and qualitative data to generate ideas, sort them according to experts’ perspectives, and use the resulting groupings to understand the priorities of a group of individuals (here, methods-focused researchers). Several limitations should be considered with our results. While the sample size met the recommendations for concept mapping studies (15), the priorities reflect the US-based sample, including many researchers at Washington University, and may not be shared among the entire D&I field. Researchers working outside of the US may have different methodological priorities given differences in geographical context. Last, the variability in the ratings, particularly for the importance variable, was fairly narrow, which makes prioritizing efforts for methods development difficult if all of the methods are considered important.

## CONCLUSIONS

This study used concept mapping to identify and organize priority areas for D&I research methods, resulting in a framework of 12 methods clusters spanning theory development, study design, measurement and analysis, stakeholder-centered approaches, and real-world application. By jointly rating methods on perceived importance and frequency of use, this work provides an empirical assessment of where the field is well equipped methodologically and where additional investment is needed to support future progress.

These findings underscore both the continued evolution and increasing coherence of D&I science. Participants articulated a broad view of “methods” that extends beyond data collection and analysis to include approaches for designing, adapting, testing, and sustaining interventions in complex, dynamic systems. The methods identified as most critical for advancing the field, including approaches to adaptation and mechanism testing, reflect areas of active methodological development rather than a lack of awareness. Differences between perceived importance and frequency of use highlight practical barriers to uptake and point to opportunities for targeted investment in training and methodological guidance.

This concept map has implications for multiple stakeholders. For researchers, it can inform methodological choices aligned with priority research questions and real-world implementation challenges. In addition to methods development through ongoing research projects, opportunities such as convenings and workshops will be important to increase the visibility of particular methods and to develop a consensus about how to develop these methods [e.g., a workshop on sustainability (63)]. For funders, including internal academic funding sources, it identifies areas where dedicated mechanisms and review criteria, for example, the use of team science, could accelerate methods development and adoption. For training and mentoring programs, it highlights underused but high-priority methods that warrant more explicit and sustained attention across career stages. By leveraging advances from complementary fields and supporting interdisciplinary team science, the D&I field can continue to strengthen its methodological foundation without duplicating existing innovations.

In summary, this study provides an empirically grounded framework to guide strategic investment in D&I methods research. Aligning methods development, training, and dissemination with these priorities will be essential for advancing rigorous, pragmatic, and equitable D&I research and for accelerating the translation of evidence into sustained improvements in practice, policy, and population-wide health.

## Supplementary Material

This is a list of supplementary files associated with this preprint. Click to download.

- SupplementalTable.docx

## Figures and Tables

**Table 1.** Sorting and Rating Participants

	N (%)
Years of experience in your field	
<5 years	8 (29%)
5–9 years	9 (32%)
10+ years	11 (39%)
D&I research expertise	
Beginner	9 (32%)
Intermediate	7 (25%)
Advanced	12 (43%)
Research methodology expertise	
Beginner	5 (18%)
Intermediate	16 (57%)
Advanced	7 (25%)
Geographic representation	
WashU-based	10 (36%)
US-based outside of WashU	13 (46%)
Internationally based	5 (18%)

**Table 2.** Summary of concept mapping results: Statement- and cluster-level importance and frequency ratings

State-ment #	Statement	Importance Rating	Frequency of Use Rating
**User-centered design**			
12	User-centered design	5.26	3.58
22	Rapid cycle qualitative methods to engage key stakeholders	4.96	3.36
30	Pragmatic approaches that allow for real world use	5.58	3.90
31	Practice-based research	5.00	4.54
50	Methods involving user or human-centered design approaches	5.07	3.50
61	Designs that consider diverse settings and cultures.	6.07	3.77
72	Assess the perspective of multiple stakeholders	5.22	4.48
	Cluster average	5.31	3.88
**Documenting and adapting implementation**			
4	Planning for adaptations of implementation and intervention strategies	5.12	3.95
18	Study designs that focus more on implementation processes, and less on implementation outcomes	4.62	4.05
28	Pragmatic measurement of organizational context	5.41	3.71
29	Pragmatic assessment of inner and outer setting factors	4.89	3.75
44	Methods/approaches to disentangle complex interventions from implementation strategies	5.07	3.43
51	Measuring adaptations of implementation and intervention strategies	5.52	3.71
59	Documenting implementation strategies in detail	5.39	3.86
85	Examination of process as well as outcome methods	5.22	4.71
89	Methods to distinguish between core specifications/attributes of intervention and those that must be adapted to context	5.07	3.32
	Cluster average	5.15	3.83
**Applied and adaptable methods**			
17	Study designs that follow programs well after initial implementation	5.52	3.52
21	Scaling-out designs	5.07	2.95
48	Methods that emphasize external validity (e.g., generalizability)	5.46	4.52
54	Iterative approaches	4.88	3.91
58	Ethnographic studies of organizational settings within which implementation is occurring.	4.77	3.22
	Cluster average	5.14	3.63
**Measurement and evaluation**			
9	Valid and reliable tools to measure implementation outcomes	5.44	4.58
10	Using core measures across studies with different innovations and different settings	5.35	3.22
46	Methods that take advantage of rich administrative data (i.e., big data)	4.56	4.09
47	Methods that make greater use of clinical administrative data, such as those from electronic health records.	4.74	4.13
49	Methods that capture organizational context	5.37	4.14
57	Evaluation of implementation outcomes	5.32	6.12
83	Greater use of organizational data	4.64	3.74
	Cluster average	5.06	4.29
**Mechanism and cost analyses**			
14	Understanding mechanisms for maintained or widened implementation disparities in care	5.56	3.13
26	Psychometrics (e.g., exploratory factor analysis [EFA], confirmatory factor analysis [CFA], varimax rotation, etc.) for developing and validating measures and instruments	5.04	3.96
32	Pooling data from several studies to ask several scientific questions	4.85	3.30
45	Methods to test mechanisms of implementation strategies	5.21	3.70
62	Cost-effectiveness studies of implementation strategies/methods	5.54	3.43
71	Assessing mechanisms (mediators, moderators)	4.93	4.05
73	Agent-based models of policy implementation	4.25	2.87
77	Mediated moderation and moderated mediation	4.11	3.50
86	Cost analyses	5.59	3.78
	Cluster average	5.01	3.52
**Partnerships and co-creation**			
19	Stakeholder engaged, participatory designs	5.31	4.13
24	Quantifying and communicating the value of partnerships with stakeholders	5.04	3.82
39	Multiple stakeholder analysis methods	4.68	3.88
65	Community-based Participatory Research	5.07	3.91
66	Community-academic partnerships	4.72	4.74
68	Co-creation with providers	4.96	3.95
69	Co-creation with policy makers and end-users	5.18	3.55
70	Co-creation with patients	4.81	3.50
	Cluster average	4.97	3.93
**Study designs**			
13	D&I research should use designs and methods from other disciplines	4.37	4.83
15	Take advantage of natural experiments.	4.81	3.50
25	Qualitative study designs and analysis (e.g., thematic content analysis, rapid analysis)	5.35	4.88
27	Prospective designs	4.60	5.50
34	Observational study designs (e.g., cross-sectional, longitudinal)	4.46	5.83
35	Observational and experimental	4.41	5.83
41	Multilevel designs	5.00	5.00
42	Mixed-methods (qualitative and quantitative)	5.81	5.08
52	Leveraging interrupted time series designs to examine retroactive or prospective policy changes using routinely collected data	4.67	3.71
79	Quasi-experimental designs, such as interrupted time series and regression discontinuity	4.79	3.83
80	Randomized controlled trials (RCTs) and variations thereof (e.g., cluster RCT, stepped-wedge RCTs, pragmatic RCTs)	4.70	6.30
82	Ethnographic and qualitative research methods	5.11	4.42
84	Longitudinal study designs	4.77	5.27
	Cluster average	4.84	4.92
**Systems and computational approaches**			
16	Systems science methods that capture heterogeneity, social structure, and system dynamics	4.74	3.35
37	Network analysis methods that can be used to assess the reach and adoption of programs across social and health systems	4.63	3.43
38	Network analysis	4.32	3.48
43	Mixed methods research that includes an explicit focus on economic evaluation	5.00	3.46
64	Computational modeling of healthcare and public health systems	4.59	3.41
75	Able to capture dynamism in the system	5.15	3.50
78	Mediational analysis	4.64	3.82
	Cluster average	4.73	3.49
**Theoretical and conceptual approaches**			
1	Logic models to separate implementation activities and outcomes from content theory	4.08	4.58
3	Use of frameworks (e.g., CFIR)	4.74	6.29
5	Mapping determinants of the implementation of programs and policies onto potential strategies for promoting implementation	5.30	4.54
7	Designing implementation strategies specifically to reduce disparities in implementation	5.64	3.00
11	Using a framework and relevant tools to collect data with participants	4.67	5.04
81	Driver diagrams to help define highest leverage change concepts to separate implementation activities and outcomes from “content theory”	3.68	3.21
	Cluster average	4.68	4.45
**Rapid cycle methods**			
23	Rapid cycle methods applied to complex interventions	4.65	3.91
55	Identifying opportunities for rapid cycle research that builds on activities already underway in practice	4.96	3.78
63	Continuous quality improvement designs	4.19	3.59
88	Rapid cycle research	4.81	3.48
	Cluster average	4.65	3.69
**Analytic methods**			
2	Causal inference (epidemiology) methods	3.93	3.59
20	Simulation methods	4.33	3.00
33	Patient-or site-level between groups comparison to assess implementation’s effect on health care disparities	5.00	3.57
36	Observation (e.g., surveys, interviews) of determinants of the implementation of evidence-based chronic disease prevention and control programs and policies	4.85	4.91
40	Multilevel methods that can capture ecological and contextual factors	5.15	4.13
56	Hierarchical or multilevel linear modeling	4.50	4.43
74	Adaptive designs (MOST, SMART)	4.96	3.57
76	Dyadic data analysis for analyses between dyads (e.g., patients-providers, providers-providers, policymakers-policymakers)	4.00	2.95
87	Longitudinal analytic methods	4.67	4.61
	Cluster average	4.60	3.86
**Designing for dissemination**			
6	Intentional use of principles of designing for dissemination	5.32	3.68
8	Consensus building methods between inner and outer settings.	3.74	3.46
53	Knowledge mobilization methods	3.58	3.24
60	Development of dissemination tools that can translate research results into accessible and actionable information for community stakeholders	5.11	3.71
67	Communications and marketing (for dissemination)	4.89	3.71
	Cluster average	4.53	3.56

## Data Availability

The datasets used and/or analyzed during the current study are available from the corresponding author on reasonable request.

## Abbreviations

D&I: Dissemination and implementation
TSBM: Translational Science Benefits Model
